# Novel Reassortant Influenza A(H5N8) Viruses among Inoculated Domestic and Wild Ducks, South Korea, 2014

**DOI:** 10.3201/eid2102.141268

**Published:** 2015-02

**Authors:** Hyun-Mi Kang, Eun-Kyoung Lee, Byung-Min Song, Jipseol Jeong, Jun-Gu Choi, Joojin Jeong, Oun-Kyong Moon, Hachung Yoon, Youngmi Cho, Young-Myong Kang, Hee-Soo Lee, Youn-Jeong Lee

**Affiliations:** Animal and Plant Quarantine Agency, Anyang, South Korea

**Keywords:** Influenza, influenza A, H5N8, HPAI, highly pathogenic avian influenza, viruses, pathogenicity, domestic duck, mallard duck, zoonoses, Eurasian eagle owl, mandarin duck, Baikal teal duck, whooper swan, spot-billed duck, sparrow hawk, common kestrel, white-fronted goose, poultry, Asia, South Korea, Republic of Korea

## Abstract

An outbreak of highly pathogenic avian influenza, caused by a novel reassortant influenza A (H5N8) virus, occurred among poultry and wild birds in South Korea in 2014. The aim of this study was to evaluate the pathogenesis in and mode of transmission of this virus among domestic and wild ducks. Three of the viruses had similar pathogenicity among infected domestic ducks: the H5N8 viruses were moderately pathogenic (0%–20% mortality rate); in wild mallard ducks, the H5N8 and H5N1 viruses did not cause severe illness or death; viral replication and shedding were greater in H5N8-infected mallards than in H5N1-infected mallards. Identification of H5N8 viruses in birds exposed to infected domestic ducks and mallards indicated that the viruses could spread by contact. We propose active surveillance to support prevention of the spread of this virus among wild birds and poultry, especially domestic ducks.

## Abstract 2

Inoculated wild ducks showed few symptoms but transmitted H5N8 viruses to other ducks.

Wild birds in orders Anseriformes (ducks, geese, swans) and Charadriiformes (gulls, terns, shore birds) are the natural reservoirs of avian influenza viruses (*1*,*2*). In wild aquatic birds, low pathogenicity avian influenza viruses are in a state of evolutionary equilibrium, and infected hosts usually show no signs of disease. However, a Qinghai-like H5N1 virus caused an outbreak in migratory waterfowl during 2005 before spreading from Asia to Europe and Africa (*3*,*4*). The outbreak gave rise to concerns that infections of wild birds with the highly pathogenic avian influenza (HPAI) virus subtype H5N1, which causes mild or no clinical signs in these birds, could result in transmission of the virus over long distances (*5*,*6*).

As was the case in other wild birds, HPAI H5N1viruses were not known to be pathogenic in domestic ducks before 2002 (*7*–*9*), but since then, HPAI H5N1 viruses that are pathogenic in ducks have been isolated in many countries (*3*,*5*,*10*,*11*). In South Korea, 4 outbreaks of HPAI H5N1have occurred among poultry (mainly chickens and domestic ducks) and wild birds. Before 2010, H5N1 HPAI viruses among birds were detected mostly in poultry (chickens, domestic ducks, and quail), with the single exception of 1 magpie in 2004. By contrast, during 2010–2011, many cases occurred in various wild birds such as the Eurasian eagle owl, mandarin duck, Baikal teal duck, mallard duck, whooper swan, spot-billed duck, sparrow hawk, common kestrel, and white-fronted goose, as well as in poultry. Although all viruses in these outbreaks were highly pathogenic in chickens, the pathogenicity of these viruses varied among domestic ducks; the pathogenicity was 0%–25% during 2003–2004 (clade 2.5, H5N1), 0% during 2006–2007 (clade 2.2, H5N1), 50%–100% during 2008 (clade 2.3.2.1, H5N1), and 50%–100% during 2010–2011 (clade 2.3.2.1, H5N1) (*5*,*12*–*15*).

Outbreaks of HPAI H5N8 infection in poultry were first reported in ducks and turkeys in Ireland in 1983. In 2010, outbreaks of infection with the HPAI H5N8 virus derived from the Goose/Guangdong/1/1996 (Gs/GD) lineage were first reported in duck farms in Jiangsu, China (*16*). Early in 2014, an outbreak of HPAI caused by a novel reassortant H5N8 virus occurred in Korea. The virus belonged to clade 2.3.4.6 and comprised 2 distinct genotypes (*17*). It has been suggested that viruses belonging to the major genotypes Buan2 and Donglim3 might be reassortants containing the polymerase basic protein 2, hemagglutinin (HA), nucleoprotein, and neuraminidase (NA) genes from viruses in the outbreak in China during 2010 (A/duck/Jiangsu/k1203/2013 (H5N8) (*17*). The HPAI H5N8 viruses were isolated from both wild birds and poultry. They were found in captured, apparently healthy, migratory wild birds and in dead birds, including mallards (both alive and dead), and in domestic chickens, geese, and ducks; the outbreak positive rate on duck farms was >70% (*13*).

There have been no previous reports about the pathogenicity of novel reassortant H5N8 isolates in wild birds and domestic ducks. Therefore, the aim of this study was to evaluate the pathogenesis in and mode of transmission of a novel reassortant H5N8 virus among mallard and domestic ducks, which are the poultry populations primarily affected by these viruses in South Korea.

## Materials and Methods

### Animals

Two species of wintering migratory birds, i.e., mallard ducks (*Anas platyrhynchos*) and Baikal teal ducks (*Anas formosa*), and 1 commercially available domestic bird (2-week-old Pekin ducks) were used in this study. The mallards and Baikal teals were captured in the wild and acquired through Konkuk University (Seoul, South Korea) and the Veterinary Epidemiology Division of the Animal and Plant Quarantine Agency. Both male and female ducks of each species were included and were approximately equally represented. All wild birds and domestic ducks used in this study were subjected to the H5 hemagglutination-inhibition (HI) test during a period of 1 week before experimentation and were maintained according to the guidelines of the Institutional Animal Care and Use Committee of Korea. All experiments were performed in a biosafety level 3–enhanced facility at the Animal and Plant Quarantine Agency, South Korea.

### Viruses

The pathogenicity of the H5N8 virus was evaluated in mallards, Baikal teals, and domestic ducks. The virus strain A/breeder duck/Kr/Gochang1/2014 (H5N8) [Gochang1] was isolated from a breeder duck in which the index case was diagnosed during the 2014 South Korean outbreak. A/broiler duck/Kr/Buan2/2014 (H5N8) [Buan2] and A/Baikal teal/Kr/Donglim3/2014 (H5N8) [Donglim3], the main strains circulating in South Korea, were isolated from a broiler duck farm and from carcasses of Baikal teals in Donglim Lake in South Korea. Two HPAI H5N1 viruses, A/chicken/Kr/IS/2006 (H5N1) (clade 2.2) [IS] and A/mandarin duck/Kr/PSC24–24/2010 (H5N1) (clade 2.3.2.1) [PSC24–24], were isolated on a chicken farm in 2006 and from a fecal sample collected from a wild bird habitat in South Korea in 2010, respectively. The viruses were propagated and titrated in specific pathogen–free (SPF) eggs and stored at −70°C until further use.

### Experimental Design

The H5N8 isolates (Gochang1, Buan2, and Donglim3) were inoculated into 38 commercially obtained 2-week-old Pekin ducks to assess their pathogenicity and transmissibility. To assess pathogenicity in mallards, we inoculated 20 captured adult males and 21 adult females with Buan2 H5N8 virus, the main genotype circulating in South Korea, or with 2 HPAI H5N1 viruses (IS and PSC24–24) circulating in South Korea in 2006 and 2010. We also assessed H5N8 pathogenicity in 2 captured adult male Baikal teals.

To test pathogenicity, we intranasally inoculated each bird with 0.1 mL of each isolate containing the 50% egg infective dose (10^6.5^ virions). After 8 h, 3 domestic ducks and 2 mallards were co-housed with inoculated birds as a contact group. We inoculated a control group of 3 domestic ducks and 2 mallards with 0.1 mL of phosphate-buffered saline using the same route. We euthanized 3 domestic birds and 2 mallards from each group 3 days postinoculation (dpi) to estimate virus recovery from various tissues.

We collected swab samples from the oropharynx and cloaca of domestic birds on 1–7, 10, and 14 dpi and from wild birds on 1, 3, 5, 7, 10, and 14 dpi. After the birds died or were euthanized, we collected tissues aseptically from the brain, trachea, lung, kidney, spleen, heart, cecal tonsil, liver, leg muscle, intestine and pancreas, and proventriculus for virus recovery. The remaining birds were observed clinically for 14 days. For virus isolation, cells from each oropharyngeal and cloacal sample were suspended in 1 mL of maintenance medium with antibiotic drugs (Antibiotic-Antimycotic; Invitrogen, Carlsbad, CA, USA), and each tissue sample was homogenized in maintenance medium with antibiotic drugs to effect a wt/vol ratio of 10%. Samples were then centrifuged at 3,500 rpm for 5 min, and 0.1 mL of supernatant was titrated in chicken embryo fibroblast cells to determine the median tissue culture infective dose (TCID_50_); virus growth was determined by observing the cytopathic effect. Virus titers were calculated as described (*18*) and the limit of virus detection was <1. We performed statistical analysis using the Student *t* test; p<0.05 was considered statistically significant.

### Serologic Assays

We collected pre-inoculation serum samples from each bird; all were confirmed to be negative for H5 HA influenza A virus by the HI assay, using standard procedures (*19*). In the H5N8 inoculation group, pre-inoculation serum samples of mallards were positive for anti-influenza virus antibody (C-ELISA; AniGen AIV Ab ELISA Kit; BioNote, Suwon, Gyeonggi-do, South Korea) but seronegative for H5 HA. Serum samples were collected from surviving wild birds, mallards, Baikal teals, and domestic ducks on 14 dpi to measure the antibody response. All serum samples were treated with receptor-destroying enzyme to remove nonspecific inhibitors (*19*).

## Results

### Clinical Signs and Mortality Rates in Domestic Ducks and Wild Birds

To determine pathogenicity, we inoculated 2-week-old domestic ducks intranasally with H5N8 viruses in groups of 3. The H5N8 viruses were moderately pathogenic (0%–20% mortality rate), and there were no differences in the pathogenicity of the 3 viruses tested. Domestic ducks inoculated with the Buan2 virus exhibited depression and neurologic signs beginning on 9 dpi; all 5 infected domestic ducks survived. Of 10 domestic ducks (in 2 groups of 5) infected with the Gochang1 and Donglim3 viruses, 1 duck died on 8 dpi and 1 died on 11 dpi, respectively. Symptoms of depression, severe weight loss (32% and 34%, respectively), cloudy eyes, and intermittent head shaking were observed before death (Table 1). In domestic ducks on 14 dpi, seroconversion rates for the Gochang1, Buan2, and Donglim3 treatment groups were 40% (5.5 ± 0.7 log_2_), 80% (7.5 ± 0.6 log_2_), and 60% (7.3 ± 0.6 log_2_), respectively. In contact-group domestic ducks, HI titers for the Gochang1 and Buan2 virus were 5 log_2_ and 6 log_2_, respectively, but none of the 3 birds in the Donglim3 group seroconverted (Table 1).

**Table 1:** Weight loss, morbidity, mortality rate, and HI titers of domestic ducks exposed to 3 strains of H5N8 influenza virus*

Virus	Group	Illness†	Weight loss, %‡	Deaths (%)	HI titer§ (log_2_, mean±SD)
A/breeder duck/Kr/Gochang1/2014 (H5N8)	Inoculated	3/5	32	1/5 (20)	2/5 (5.5 ± 0.7)
	Contact¶	2/3	21	0/3	1/3 (5)
A/broiler duck/Kr/Buan2/2014 (H5N8)	Inoculated	2/5	28	0/5	4/5 (7.5 ± 0.6)
	Contact¶	1/3	26	0/3	1/3 (6)
A/Baikal teal/Kr/Donglim3/2014 (H5N8)	Inoculated	3/5	34	1/5 (20)	3/5 (7.3)
	Contact¶	2/3	28	0/3	0/3
Controls (no virus exposure)		0/3	–	0/3	0/3

*Unless indicated otherwise, data represent the number of affected animals/ animals in the group. Animals were inoculated by the intranasal route with 10^6.5^ egg-infective dose_50_/0.1 mL of the selected viruses; HI, hemagglutination inhibition. †Severe depression, cloudy eye, and intermittent head-shaking. ‡Weight loss was measured at 11 d postinoculation and is expressed as a percentage of the weight of preinoculation ducks. §HI titer was assayed in serum samples taken at 14 d postinoculation. Data show the ratio of antibody-positive animals to the number of virus-inoculated animals. ¶Three uninoculated birds were co-housed with infected birds as a contact group 8 h after inoculation.

Mallards excrete abundant quantities of HPAI H5N1 virus without exhibiting clinical signs of disease (*10*,*20*). In this study, none of the mallards infected with H5N1 or H5N8 viruses died. They exhibited mild or no symptoms after inoculation with the H5N8 or H5N1 virus. In mallards that were inoculated with H5N8, or in those that were in contact with H5N8 virus–inoculated birds, HI titers were much higher than those observed for the 2 H5N1 viruses. Of 5 mallards inoculated with the H5N8 virus, 4 (80%) seroconverted and showed high titers (9.0 ± 0.8 log_2_), and 2 contact-group ducks seroconverted with relatively high titers (7.0 ± 2.8 log_2_). The HI titers of groups inoculated with IS and PSC24–24 H5N1 viruses were 5.0 ± 0.7 log_2_, and 4 log_2_, respectively. In the IS and PSC24–24 virus-contact groups, the seroconversion rates were 50% (5 log_2_) and 0%, respectively.

Unlike mallards, 1 of the Baikal teals inoculated with an H5N8 virus died suddenly on 3 dpi without clinical symptoms. The surviving Baikal teal seroconverted to H5N8 with a relatively high titer (6 log_2_) (Table 2).

**Table 2:** Virus isolation from swab samples obtained from 2 Baikal teal ducks inoculated with influenza A/broilerduck/Kr/Buan2/2014 (H5N8) virus*

Virus titer, log_10_ TCID_50_/0.1 mL (no. positive/no. inoculated)†																			HI titer‡
Oropharyngeal samples, days postinoculation										Cloacal samples, days postinoculation									
1	2	3	4	5	6	7	10	14		1	2	3	4	5	6	7	10	14	
4.5 (1/2)	4.2 (1/2)	0 (0/1)	0 (0/1)	0 (0/1)	0 (0/1)	0 (0/1)	0 (0/1)	0 (0/1)		0 (0/2)	1.2 (1/2)	0 (0/1)	0 (0/1)	0 (0/1)	0 (0/1)	0 (0/1)	0 (0/1)	0 (0/1)	1/1

*Birds were inoculated with 10^6.5^ 50% egg-infective dose/0.1 mL of virus via the intranasal route, and oropharyngeal and cloacal swab samples were collected on the indicated day. No clinical signs of illness were observed in the birds; however, 1 bird died on day 3 post-inoculation; TCID, tissue culture infective dose; HI, hemagglutination inhibition. †Virus was detected only in the bird that died. ‡HI titer was assayed in a serum sample taken at day 14 postinoculation; data shows the ratio of the number of antibody-positive animals to the number of virus-inoculated animals.

### Replication in and Transmission among Domestic Ducks

The phenotypes of 3 H5N8 viruses, which were observed in 2 genotypes (Buan2/Donglim3, and Gochang1), were evaluated in domestic ducks. We found significant differences in the viral shedding of 3 H5N8 viruses in domestic ducks between Buan2 and Donglim3 viruses on cloacal swab samples on 5 dpi (p<0.05). In tissues, Donglim3 viral titers from trachea and lung samples were significantly higher than Buan2 virus titers, whereas Buan2 viral titers from spleen samples were significantly higher than those of Donglim3 (p<0.05), according to the results of the Student *t* test. The virus was not detected in a control group of domestic ducks (data not shown) that were not inoculated. In the infected domestic ducks, Gochang1 was recovered from the oropharynx (10^1.3–4.4^ TCID_50_/0.1 mL) on 1–7 dpi and from the cloaca (10^0.6–3.6^ TCID_50_/0.1 mL) on 1–6 dpi. The Buan2 virus was re-isolated from the oropharynx (10^0.6–3.7^ TCID_50_/0.1 mL) on 1–10 dpi and from the cloaca (10^0.6–2.9^ TCID_50_/0.1 mL) on 1–5 dpi. Donglim3 was recovered from the oropharynx (10^1.1–4.5^ TCID_50_/0.1 mL) on 1–10 dpi and from the cloaca (10^0.6–3.4^ TCID_50_/0.1 mL) on 2–7 dpi (Figure). The H5N8 viruses were replicated systemically in, and re-isolated from, various tissues of domestic ducks with titers that varied from 10^0.7^ to 10^7.6^ TCID_50_/0.1 mL.

**Figure:**
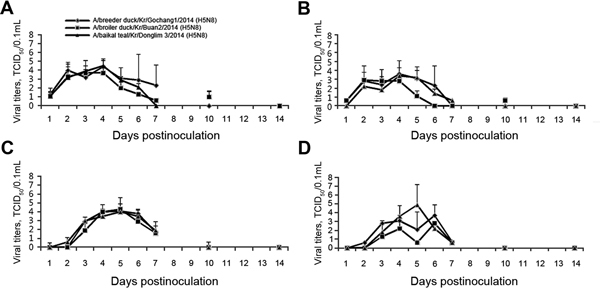
Virus isolation from oropharyngeal (OP) or cloacal (CL) swab samples collected from domestic ducks exposed to influenza viruses by inoculation or contact with infected ducks. Nine ducks were intranasally inoculated with 10^6.5^ egg infectious dose titer of A/breeder ducks/Kr/Gochang1/2014 (H5N8), A/broiler duck/Kr/Buan2/2014 (H5N8), or A/Baikal teal/Kr/Donglim3/2014 (H5N8) viruses (A and B). Six domestic ducks that were not inoculated were co-housed with 3 contact groups (2 in each group) of infected ducks (C and D). TCID_50_, 50% tissue culture infectious dose. Error bars indicate SD.

Unlike the other 2 H5N8 viruses, Gochang1 replicated at low titers (10^1.6^ TCID_50_/0.1 mL) in brain and other tissues. Gochang1 and Donglim3 viruses were isolated from several tissues of a dead inoculated bird (Table 3).

**Table 3:** Virus titers in tissues of domestic ducks and mallard ducks inoculated intranasally with H5N8 and H5N1 influenza viruses

Host species, virus	No. ducks, mode of death*	dpi	Virus isolation titer in tissue samples (no. positive/no. ducks)†										
Tra	Liv	Mus	Pro	Int, Pan	Spl	Ct	Lung	Kid	Heart	Brain
Domestic ducks													
A/breeder duck/Kr/Gochang1/2014 (H5N8)	3, euthanized	3	6.7 ± 1.1 (3/3)	1.5 ± 0.4 (2/3)	1.0 ± 0.6 (2/3)	2.6 ± 1.6 (3/3)	0.9 ± 0.4 (2/3)	2.4 ± 0.9 (3/3)	2.8 ± 0.5 (2/3)	4.2 ± 0.5 (3/3)	3.2 ± 0.7 (3/3)	2.4 ± 0.6 (3/3)	1.6 ± 1.3 (2/3)
	1, died	10	3.8	0	0	2.5	1.5	0	2.4	3.9	0	3.5	4.2
A/broiler duck/Kr/Buan2/ 2014 (H5N8)	3, euthanized	3	4.7 ± 1.3 (3/3)	2.2 ± 0.4 (3/3)	1.9 ± 1.3 (3/3)	2.8 ± 0.5 (3/3)	0 (0/3)	3.3 ± 0.6^‡^ (2/3)	2.0 ± 0.8 (2/3)	3.5 ± 0.1 (3/3)	2.3 ± 0.9 (3/3)	2.2 ± 0.6 (3/3)	0 (0/3)
A/Baikal teal/Kr/Donglim3/ 2014 (H5N8)	3, euthanized	3	7.6 ± 0.9‡ (3/3)	2.4 ± 1.2 (2/3)	1.9 ± 0.7 (2/3)	1.9 ± 0.9 (3/3)	1.8 (1/3)	0.7 ± 0.1 (2/3)	2.3 ± 0.1 (2/3)	4.5 ± 0.1^‡^ (3/3)	2.8 ± 0.8 (3/3)	1.6 ± 0.8 (3/3)	4.0 (1/3)
	1, died	10	0	0	0	0	1.5	1.5	0	0	0	0	0
Control													
NI	2, euthanized	3	0 (0/2)	0 (0/2)	0 (0/2)	0 (0/2)	0 (0/2)	0 (0/2)	0 (0/2)	0 (0/2)	0 (0/2)	0 (0/2)	0 (0/2)
Mallard ducks													
A/broiler duck/Kr/Buan2/ 2014 (H5N8)	2, euthanized	3	2.6 (1/2)	0 (0/2)	2.2 (1/2)	2.2 ± 1.9 (2/2)	2.2 ± 0.5 (2/2)	0 (0/2)	1.8 (1/2)	1.9 ± 0.7 (2/2)	2.5 ± 1.8 (2/2)	2.2 (1/2)	0 (0/2)
A/chicken/Kr/IS/ 2006 (H5N1)	2, euthanized	3	0 (0/2)	0 (0/2)	0 (0/2)	0 (0/2)	0 (0/2)	0 (0/2)	0 (0/2)	0 (0/2)	0 (0/2)	0 (0/2)	0 (0/2)
A/mandarin duck/Kr/PSC24–24/2010 (H5N1)	2, euthanized	3	0 (0/2)	0 (0/2)	0 (0/2)	0 (0/2)	0 (0/2)	0 (0/2)	0 (0/2)	0 (0/2)	0 (0/2)	0 (0/2)	0 (0/2)

*Tissues were collected from euthanized birds or birds that died from the virus on 3 dpi. dpi, days post inoculation; log_10_ TCID_50_, tissue culture infective dose; Tra, trachea; Liv, liver; Mus, muscle; Pro, proventriculus; Int, intestine; Pan, pancreas; Spl, spleen; Ct, cecal tonsil; Kid, kidney, NI, control ducks were not inoculated. †Virus titers in tissues from domestic ducks inoculated intranasally with 10^6.5^ 50% egg-infective dose of H5N8 influenza virus; values are mean+SD TCID titers/0.1 mL. ‡Donglim3 viral titers were significantly higher than those of Buan2 virus from trachea and lung (p<0.05), whereas Buan2 viral titers from spleen were significantly higher than those of Donglim3 (p<0.05).

In domestic contact ducks, all 3 H5N8 viruses were recovered in swab samples, indicating that the H5N8 viruses could have spread by contact. Gochang1 virus was recovered from the oropharynx (10^1.7–4.1^ TCID_50_/0.1 mL) on 3–7 dpi and from the cloaca (10^0.6–3.7^ TCID_50_/0.1 mL) on 2–7 dpi. The Buan2 virus was recovered from the oropharynx (10^1.6–4.3^ TCID_50_/0.1 mL) on 3–7 dpi and from the cloaca (10^0.6–2.2^ TCID_50_/0.1 mL) on 3–7 dpi. Likewise, Donglim3 virus was recovered from the oropharynx (10^0.6–4.0^ TCID_50_/0.1 mL) on 2–7 dpi and from the cloaca (10^0.6–4.9^ TCID_50_/0.1 mL) on 3–7 dpi.

### Virus Replication in and Transmission among Wild Birds

The extent of replication and transmissibility of a virus in the host animal has a major influence on the magnitude of outbreaks. To evaluate the pathogenicity of the Buan2 H5N8 virus in comparison to that of 2 H5N1 viruses (IS06 and PSC24–24), mallards were inoculated intranasally with the viruses. H5N8 virus was re-isolated from the oropharynx (10^1.0–3.4^ TCID_50_/0.1 mL) on 1–5 dpi and from the cloaca (10^2.7^ TCID_50_/0.1 mL) on 3 dpi. In the H5N1-infected groups, the viruses were recovered from the oropharynx on 1–3 dpi, (10^1.8–2.0^ TCID_50_/0.1 mL) but not from the cloaca. The titers of the IS06 and PSC24–24 H5N1 virus re-isolated from oropharyngeal samples were significantly lower than that of the H5N8 virus on 3 dpi (p<0.01) (Table 4). To determine whether the HPAI viruses can be efficiently transmitted among mallards, we performed the virus isolation procedures using oropharyngeal and cloacal samples obtained from mallards in the contact groups. All 3 H5 viruses were recovered, but their shedding patterns varied. H5N8 virus was recovered from the oropharynx (10^2.2–2.5^ TCID_50_/0.1 mL) on 3–5 dpi and from the cloaca (10^0.6^ TCID_50_/0.1 mL) on 3 dpi. However, the 2 H5N1 viruses could only be re-isolated from the oropharynx at low titers (10^1.8–2.0^ TCID_50_/0.1 mL) (Table 4).

**Table 4:** Virus isolation from swab samples obtained from mallard ducks inoculated with H5N8 and H5N1 influenza viruses*

Virus	Group	No. dead/no inoculated	Clinical signs	Virus titer, log_10_TCID_50_/0.1 mL, mean±SD (no. positive/no. ducks)†														HI titer
				Oropharyngeal swab sample results, (days post-inoculation)								Cloacal swab sample results (days post-inoculation)							
				1	3		5	7	10	14		1	3	5	7	10	14		
A/broiler duck/Kr/Buan2/2014 (H5N8)	Inoculated	0/5	None	1.0 ± 0.5 (4/7)		3.4 ± 0.6 (6/7)	1.5 (1/5)	0 (0/5)	0 (0/5)	0 (0/5)		0 (0/7)	2.7 ± 1.2 (2/7)	0 (0/5)	0 (0/5)	0 (0/5)	0 (0/5)	4/5	
	Contact‡	0/2	None	0 (0/2)	2.2 ± 0.9 (2/2)		2.5 (1/2)	0 (0/2)	0 (0/2)	0 (0/2)		0 (0/2)	0.6 (1/2)	0 (0/2)	0 (0/2)	0 (0/2)	0 (0/2)	2/2	
A/chicken/Kr/IS/2006 (H5N1)	Inoculated	0/2	Mild§	1.8 (1/4)	2.0 ± 0.2¶ (4/4)		0 (0/2)	0 (0/2)	0 (0/2)	0 (0/2)		0 (0/4)	0 (0/4)	0 (0/2)	0 (0/2)	0 (0/2)	0 (0/2)	2/2	
	Contact	0/2	None	0 (0/2)	1.8 (1/2)		0 (0/2)	0 (0/2)	0 (0/2)	0 (0/2)		0 (0/2)	0 (0/2)	0 (0/2)	0 (0/2)	0 (0/2)	0 (0/2)	1/2
A/mandarin duck/Kr/PSC24–24/2010 (H5N1)	Inoculated	0/2	Mild	0 (0/4)	2.0 ± 1.2 (4/4)		0 (0/2)	0 (0/2)	0 (0/2)	0 (0/2)		0 (0/4)	0 (0/4)	0 (0/2)	0 (0/2)	0 (0/2)	0 (0/2)	1/2	
	Contact	0/2	Mild	0 (0/2)	2.0 ± 0.8 (2/2)		0 (0/2)	0 (0/2)	0 (0/2)	0 (0/2)		0 (0/2)	0 (0/2)	0 (0/2)	0 (0/2)	0 (0/2)	0 (0/2)	0/2	
Control	NA	0/2	None	0 (0/2)	0 (0/2)		0 (0/2)	0 (0/2)	0 (0/2)	0 (0/2)		0 (0/2)	0 (0/2)	0 (0/2)	0 (0/2)	0 (0/2)	0 (0/2)	0/2	

*Birds were inoculated intranasally with 10^6.5^ egg-infective dose/0.1 mL of virus; oropharyngeal and cloacal swab samples were taken on the indicated day; HI, hemagglutination inhibition titer. HI titer was assayed in serum taken at 14 days post-inoculation. Data show the ratio of the number of antibody-positive animals to the number of virus-inoculated animals. †Virus titer is an average of the virus titers of positive samples. ‡A pair of control birds were co-housed with the infected birds as a contact group at 8 h after inoculation. §Mild clinical signs, diarrhea or cloudy eye. ¶Significant differences in oropharyngeal shedding between Buan2 and IS06 on day 3 post-inoculation (p<0.01) as estimated by the Student *t*-test.

The H5N8 virus was isolated from tissues collected from euthanized mallards on 3 dpi. It was replicated systemically in the trachea, muscle, proventriculus, intestine (pancreas), cecal tonsil, lung, kidney, and heart, and was present at low titers (10^1.8–2.6^ TCID_50_/0.1 mL). In infected birds, H5N1 IS06 and PSC24–24 viruses did not replicate in any of the tissues tested. H5N1 viruses were not detected in mallardsin uninoculated control groups (Table 3).

Two Baikal teal ducks were inoculated with influenza A/broiler duck/Kr/Buan2/2014 (H5N8). One of the ducks died on 3 dpi. H5N8 virus was re-isolated from its oropharyngeal sample (10^4.2–4.5^ TCID_50_/0.1 mL) on 1 and 2 dpi and from the cloacal sample (10^1.2^ TCID_50_/0.1 mL) on 2 dpi (Table 2). The virus had replicated efficiently in the tissues of the bird by 3 dpi, and investigation after its death showed titers of 10^2.4^ TCID_50_/0.1 mL in the trachea, 10^1.3^ in muscle, 10^8.4^ in liver, 10^2.6^ in the proventriculus, 10^3.2^ in the intestine (pancreas), 10^2.6^ in the spleen, 10^2.8^ in the cecal tonsil, 10^2.4^ in the lung, 10^1.6^ in the heart, and 10^3.2^ in the kidney (data not shown). No clinical signs of disease were observed in either duck.

## Discussion

In a previous study, we reported the first influenza outbreak in South Korea in poultry and wild birds caused by a novel reassortant H5N8 virus in 2014 (*13*). The virus was composed of 2 distinct genogroups (*17*), and the most affected poultry species was the domestic duck at a 75.5% infection rate. Many H5N8 isolates were obtained from dead wild birds, but some were obtained from live wild birds, including mallards. In this study, we evaluated the pathogenicity of these novel reassortant HPAI H5N8 viruses in wild mallard and young domestic ducks.

The clade 2.3.4, which is the major genotype in China, continues to evolve as subclades, resulting thus far in 2.3.4.1, 2.3.4.2, 2.3.4.3, 2.3.4.4, 2.3.4.5, and 2.3.4.6 (*21*). In addition, clade 2.3.4 appears in various NA subtypes such as H5N5, H5N8, and H5N2 (*22*,*23*). The pathogenicity of H5 viruses with different NA subtypes has been evaluated. A mortality rate of 50% in 4-week-old ducks was attributed to H5N5 viruses isolated from ducks in live bird markets of China in 2008 (*24*). The pathogenicity of H5N5 and H5N8 viruses isolated from poultry in China during 2009–2010 varied from mild to moderate among mallards (*16*). The pathogenicity of H5N8 viruses isolated from domestic ducks in eastern China in 2013, which was similar to that of the Gochang1 virus described here, has been evaluated only in chickens and mice (*16*).

We selected 3 H5N8 viruses belonging to 2 distinct genotypes (*13*) from different host animals and evaluated their pathogenicity in 2-week-old domestic ducks. There were no differences in the pathogenicity of the 3 H5N8 viruses found in South Korea, and they were less pathogenic (0%–20% mortality rate) than previous H5N1 viruses in South Korea that caused outbreaks in 2008 and 2010 (50%–100% mortality rate) (*10*,*12*,*25*). However, H5N8 viral shedding by domestic ducks was much greater (10^4.5^ TCID_50_/0.1 mL) in both oropharyngeal and cloacal swab samples than shedding of H5N1 viruses found in South Korea during 2008 (10^3.8^ TCID_50_/0.1 mL) and 2010 (10^2.8^ TCID_50_/0.1 mL) (*12*,*25*). The ability of these novel reassortant H5N8 viruses to replicate efficiently in the respiratory and intestinal tracts without killing the infected ducks enables them to circulate within the duck population and increases the possibility of transmission on poultry farms. Indeed, clinical signs and death were rare during the 2014 H5N8 outbreak in South Korea, except for a drop in egg production at duck breeder farms. The results of this study suggest that domestic ducks may be silent carriers of novel reassortant H5N8 viruses, which may make it difficult to detect these viruses in domestic duck farms or live bird markets. Moreover, the efficient replication, high seroconversion, and shedding of relatively high titers in the contact groups suggest that the H5N8 virus was efficiently transmitted among ducks. Therefore, active surveillance designed to detect infection, especially in domestic ducks, should be enforced on farms and at live bird markets.

Tang et al. (*26*) suggested that certain amino acid substitutions (Q→L at position 9) and 1 basic amino acid deletion within the HA cleavage site may be consequential for H5N1 pathogenicity in ducks. As mentioned previous report (*17*), each of the 3 H5N8 viruses in our study showed L at position 9 with a deletion at position 4 of the HA cleavage site, whereas H5N1 viruses circulating in South Korea in 2008 and 2010 showed Q at position 9 with a deletion at the same position (*5*,*12*). However, previous H5N1 viruses were reported as more pathogenic (50%–100%) than H5N8 viruses (0%–20%) in studies that used domestic ducks as hosts (*10*,*12*,*25*). Our results suggest that molecular factors other than amino acid substitutions in the HA cleavage site may be involved in the pathogenicity of these viruses in domestic ducks. Further study is required to identify the molecular factors determining virus pathogenicity in domestic ducks.

Some of the H5N1 and H5N8 viruses were isolated from apparently healthy mallards captured in South Korea during the 2010–11 (*5*) and 2014 outbreaks. We selected 1 H5N8 and 2 H5N1 viruses to evaluate their pathogenicity and transmission in mallards. Although severe morbidity or mortality rates were not observed in the mallards inoculated with H5N8 or H5N1 viruses or in those housed with inoculated ducks, viral shedding and replication in tissues were higher and the duration of viral shedding was longer in mallards infected with H5N8 virus than in those infected with H5N1 virus. In this study, the significant shedding of H5N8 viruses (p<0.05) by mallards is consistent with the hypothesis that mallards may be long distance vectors of these viruses, as was observed for HPAI H5N1 (*20*,*27*). Moreover, a significant difference in the viral shedding was found between domestic ducks and mallards: the Buan2 viral titers in domestic ducks were significantly higher than those in mallards on oropharyngeal and cloacal swabs on 2 and 4 dpi. (p < 0.05). Our results suggest that the novel reassortant H5N8 virus replicated more efficiently than H5N1 viruses in mallards and that efficient horizontal transmission occurred, resulting in the transmission of influenza virus between mallards and facilitating the spread of influenza viruses among wild birds.

The Baikal teal reproduces in eastern Siberia, Russia, and flies over Mongolia and North Korea. It winters mainly in Japan and South Korea, which now host the majority of the wintering population, along with the mallards (*Anas platyrhynchos*) and white-fronted goose (*Anser albifrons*). Although the number of the Baikal teals in our study was small, the results are consistent with field observations during the 2014 H5N8 outbreak in South Korea during which many H5N8 viruses were isolated from carcasses of Baikal teals or captured H5-seropositive Baikal teals (data not shown).

Many of the H5N1 viruses that emerged after 2002 had a particularly high level of virus shedding from tracheal samples (*11*,*14*,*20*,*28*,*29*). In our study, viral titers and shedding duration were relatively high in both oropharyngeal and cloacal swab samples from domestic ducks and mallards inoculated with H5N8 viruses (Table 4), indicating that H5N8 viruses could be efficiently transmitted through the respiratory or respiratory-digestive tract (*30*).

In conclusion, a novel reassortant H5N8 virus circulating in South Korea in 2014 displayed moderate pathogenicity with efficient viral shedding and replication in tissues of domestic ducks. Although infection with H5N8 and H5N1 viruses did not result in severe morbidity or mortality rates, viral shedding and replication in tissues were higher in mallards infected with H5N8 than in those infected with H5N1 viruses. Moreover, the H5N8 viruses were recovered from uninoculated domestic and mallard ducks that were in contact with infected birds, indicating efficient horizontal transmission. Our findings emphasize the need to expand active surveillance to help prevent the spread of this virus among wild birds and poultry, especially domestic ducks.
